# Effects of Oxygen Adsorption on the Optical Properties of Ag Nanoparticles

**DOI:** 10.1021/acs.jpca.3c05801

**Published:** 2023-12-01

**Authors:** Elena Zerbato, Riccardo Farris, Giovanna Fronzoni, Konstantin M. Neyman, Mauro Stener, Albert Bruix

**Affiliations:** † Dipartimento di Scienze Chimiche e Farmaceutiche, Università di Trieste, Via L. Giorgieri 1, Trieste 34127, Italy; ‡ Departament de Ciència del Materials i Química Física & Institut de Química Teòrica i Computacional, Universitat de Barcelona, Barcelona 08028, Spain; § ICREA (Institució Catalana de Recerca i Estudis Avançats), Barcelona 08010, Spain

## Abstract

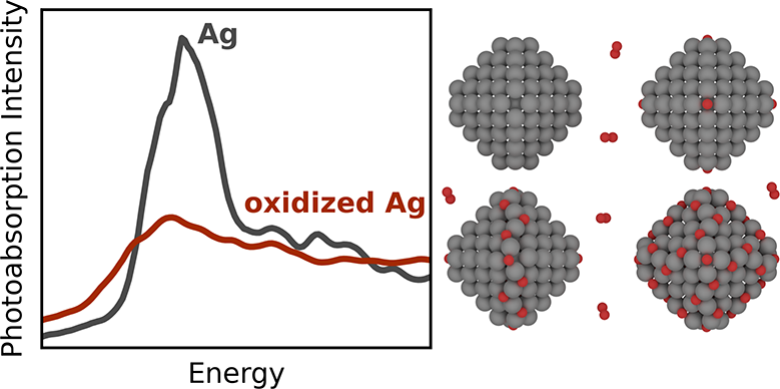

Plasmonic metal nanoparticles are efficient light harvesters with a myriad of sensing- and energy-related applications. For such applications, the optical properties of nanoparticles of metals such as Cu, Ag, and Au can be tuned by controlling the composition, particle size, and shape, but less is known about the effects of oxidation on the plasmon resonances. In this work, we elucidate the effects of O adsorption on the optical properties of Ag particles by evaluating the thermodynamic properties of O-decorated Ag particles with calculations based on the density functional theory and subsequently computing the photoabsorption spectra with a computationally efficient time-dependent density functional theory approach. We identify stable Ag nanoparticle structures with oxidized edges and a quenching of the plasmonic character of the metal particles upon oxidation and trace back this effect to the *sp* orbitals (or bands) of Ag particles being involved both in the plasmonic excitation and in the hybridization to form bonds with the adsorbed O atoms. Our work has important implications for the understanding and application of plasmonic metal nanoparticles and plasmon-mediated processes under oxidizing environments.

## 1 Introduction

Metal nanoparticles (NPs) exhibit intriguing electronic and optical^1−3^ properties, which make them ideal building blocks of next-generation electronic, optoelectronic, and chemical sensing devices. Metal NPs are also extensively used in catalysis, playing a key role in the chemical industry and energy-related applications.^4^ Despite their numerous and promising applications, a full understanding of the physical and chemical properties of these systems at the nanoscale is still needed to enable tunability and actual implementation. A notable example of optical properties at the nanoscale is the striking changes of color from macroscopic (bulk) metals to metal NPs. This is the case of gold NPs, which exhibit a range of colors between red and blue depending on the particle size. These peculiar properties can be traced back to an intricate interplay between surface effects^5^ and quantum-size effects.^6,7^

Optical properties of metal particles have long been a subject of interest in physical chemistry, beginning with Faraday^8^ and Mie^9,10^ relying on Maxwell’s electromagnetism. For particles smaller than ∼10 nm, quantum effects start to govern the optical response. The optical properties of Au, Ag, and Cu NPs are mainly due to the localized surface plasmon resonance (LSPR). For silver and gold nanoparticles, the resonance falls within the visible region of the electromagnetic spectrum, for which they display striking.^11−13^ The LSPR consists of damped yet collective oscillations of conduction electrons in a metal nanoparticle triggered by the interaction with an electromagnetic field, e.g., visible light. Thanks to this behavior, plasmonic metal NPs are efficient light harvesters, where their tunable light-matter interactions give rise to applications in photodetection,^14^ photocatalysis,^15^ solar energy conversion,^16^ and related fields.^17^ Whereas the effect of NP size,^18−21^ shape,^22^ and composition^23,24^ on the resulting optical properties has been extensively explored in the literature, how the oxidation of Cu, Au, and Ag NPs affects their optical properties is less clear.^25^ Schira and Rabilloud^26^ have investigated how the adsorption of up to three O atoms affects the photoabsorption spectrum of small Ag particles, but the effects of oxidation on larger crystalline nanoparticles have not yet been addressed.

Oxygen adsorption and dissociation on metals are one of the key stages of many industrially relevant catalytic reactions and corrosion processes. Depending on the environmental conditions and the thermodynamics of the process, the oxidation of transition metals may result in their surfaces decorated with a few coadsorbed atoms, the full oxidation to the corresponding metal oxide, or something in between. The latter refers to the formation of an oxide overlayer, i.e., surface oxides or passivated surfaces.^27,28^ Although Au is generally quite resistant to oxidation, Ag and Cu can more easily become partially or completely oxidized.^29−32^ It is therefore necessary to evaluate how such facile oxidation processes affect the optical properties of plasmonic metal NPs for applications in atmospheric or other O-rich environments. Furthermore, Ag is rarely used as a stand-alone photocatalyst and is instead typically combined (as a cocatalyst) with active photocatalytic materials such as TiO_2_ to improve charge separation and overall performance.^33−36^ Characterizing the effects of Ag oxidation, which can also result from the interactions of metal particle on reducible oxide supports,^37^ is therefore also crucial to understand the photoactivity of Ag NPs in such composite materials.

In part because of the catalytic applications of Ag-based materials in epoxidation reactions,^38^ the reactivity of Ag surfaces and nanostructures toward O_2_ has been extensively addressed both experimentally^38−41^ and in computational studies.^42−45^ The first relevant study in this regard can be traced back to that of Rovida et al. in 1972,^46^ where adsorption of oxygen over Ag(111) was described.^32,47^ When scaling down from metal surfaces to metal nanoparticles, size effects and effects related to the presence of more undercoordinated metal sites emerge, making the dissociation of O_2_ more facile.^48,49^ However, it is challenging to determine which are the stable equilibrium structures of oxidized Ag systems; the structure and chemical state of Ag surface oxides have been actively investigated and discussed,^29,31,32^ but for Ag NPs, little is known.

To address the above-mentioned challenges, in this work, we elucidate the effects of oxidation on the optical properties of Ag particles by evaluating the thermodynamic stability of O-decorated Ag particles by means of calculations based on the density functional theory (DFT) and subsequently computing their photoabsorption spectra with a computationally efficient time-dependent density functional theory (TDDFT) approach.^50^ We thus identify a quenching of the plasmonic character of the metal particles upon oxidation and trace back this effect to the fact that the *sp* orbitals (or bands) of Ag particles are involved both in the plasmonic excitation and in the hybridization to form bonds with the adsorbed O atoms.

## 2 Theoretical Methods and Computational Details

### 2.1 Structural Models of Ag NPs

**Figure 1:**
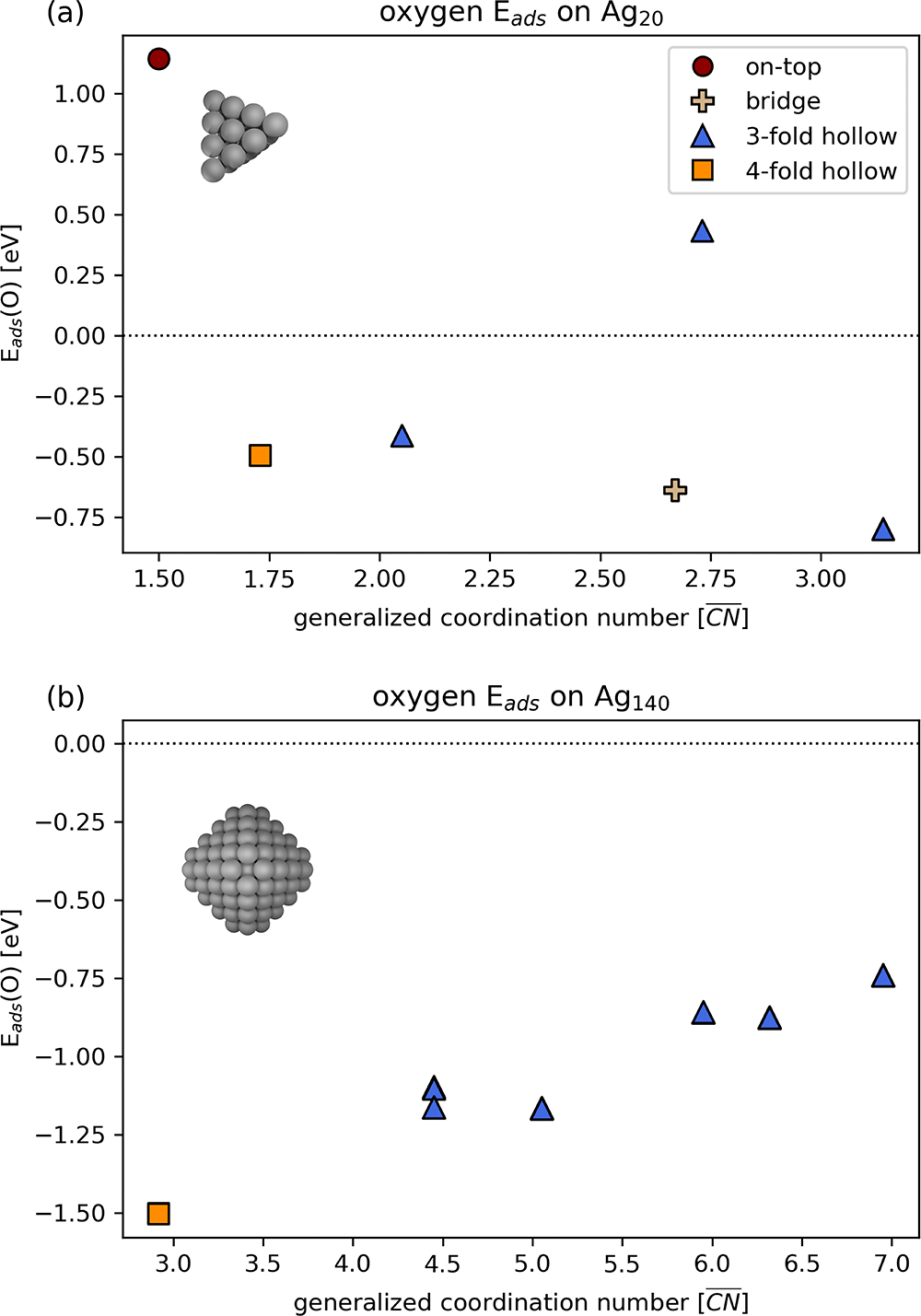
Adsorption energies *E*_ads_(O) of single O atoms on different sites of Ag_20_ (a) and Ag_140_ (b) nanoparticles as a function of the generalized coordination number of the adsorption site. The corresponding relaxed structures are illustrated in Figure S1, and the values are tabulated in Tables 1 and 2

As models for Ag NPs, we used a tetrahedral Ag_20_ particle and a truncated octahedral Ag_140_ particle (Figure 1, insets). The tetrahedral Ag_20_ particle was shown to exhibit a single photoabsorption peak with high oscillation strength^18^ and is therefore an ideally small probe system to evaluate the stability of many O_N_/Ag_20_ structures and benchmark the employed TDDFT approach. The larger Ag_140_ NP is, in turn, more representative of larger Ag particles used in applications with well-defined facets and bulk region.

### 2.2 Geometry Optimizations

Geometry optimizations for the bare and O-decorated Ag particles were performed using the plane-wave code Vienna Ab Initio Simulation Package (VASP).^51,52^ The gradient-corrected Perdew–Burke–Ernzerhof (PBE)^53^ exchange–correlation functional was employed, which showed a good balance between computational cost and reliability for describing bulk, surface, and chemical properties of transition metals.^54,55−57^ The kinetic energy cutoff for the plane-wave basis set expansion was set at 415 eV, and the projector augmented wave^58,59^ approach was used to describe the interaction between core electrons and valence electrons. The Brillouin zone was sampled at the Γ-point. One-electron Kohn–Sham (KS) levels were smeared by 0.1 eV, and the converged total energies were finally extrapolated to zero smearing. All atoms were locally relaxed without any constraints until forces on each atom were smaller than 0.01 eV/Å. Projected density of states was extracted from the corresponding VASP DOSCAR files. The preparation of inputs and the analysis of outputs were carried out with the Atomic Simulation Environment^60^ and our own NanoPartlicleLibrary python package.^61^

### 2.3 Adsorption Energies

The adsorption energy, *E*_ads_(O), of oxygen atoms on different sites of silver nanoparticles was calculated as
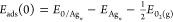
1where *E*_O/Ag_*n*__, *E*_Ag_*n*__, and *E*_O_2_(g)_ represent the total DFT energy of the Ag_*n*_ NP with an adsorbed oxygen atom, the bare Ag_*n*_ NP without the O atom, and the gas-phase O_2_ molecule in its triplet ground state, respectively. Negative adsorption energies thus correspond to exothermic adsorptions. To compute the adsorption energies for *N* > 1 oxygen atoms, incremental adsorption energies, Δ*E*_ads_*(*O), were calculated as
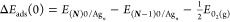
2where *N* is the number of adsorbed O atoms. Incremental adsorption energies are suitable to effectively evaluate whether the progressive adsorption of oxygen atoms is thermodynamically favored. They allow the identification of the O-saturation coverages.

For some selected cases with either molecularly adsorbed O_2_ or two coadsorbed O atoms, the adsorption energy of an O_2_ molecule, *E*_ads_(O_2_), was calculated as
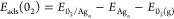
3

### 2.4 Photoabsorption Spectra Calculations

The typical TDDFT implementation in conventional quantum chemistry consists of the linear combination of atomic orbitals (LCAO) according to the Casida formalism.^62^ This combines the reliability of TDDFT approaches to describe electron excitations with the accuracy of the LCAO implementation, avoiding the drastic approximation of the effective potential intrinsic in the simplified jellium approach.^63^ For example, optical spectra of metal clusters protected by ligands have been calculated via TDDFT using a finite basis set of contracted Gaussian-type orbitals^64,65^ or a plane-wave basis set.^66^ From the computational point of view, the Casida method consists of diagonalizing, by means of a Davidson algorithm, a matrix whose dimensions correspond to all the pairs of occupied and virtual orbitals (*N*_occ_ × *N*_virt_). The photoabsorption spectrum is then obtained from eigenvalues (squared excitation energies) and eigenvectors (intensities). Moreover, an analysis of the eigenvectors of the Casida matrix allows one to completely assign the electronic transitions in terms of the electronic structure, as each transition is described as a linear combination of one-electron excited electron/hole (1h1p) configurations. It is worth noting that the Davidson diagonalization is very efficient and can be applied to very large matrices, but only a small number of the lowest eigenvalues/eigenvectors can be extracted. Therefore, only a narrow interval of the absorption spectrum can be calculated in practice for large systems. Wider intervals of the absorption spectrum can be obtained by using an alternative TDDFT algorithm that extracts the spectrum from complex polarizability. One such algorithm is the polTDDFT method,^50^ which we have employed in this work.

Photoabsorption spectra were thus obtained by means of TDDFT calculations within the AMS/ADF^67^ program suite version 2021.106. In all calculations, the LB94^68^ exchange–correlation potential was employed to obtain KS orbitals and eigenvalues from KS equations. The latter potential was introduced by Van Leeuwen and Baerends proved to be particularly suited for TDDFT calculations, as it exhibits the correct Coulombic asymptotic decay at long range, ∝ – 1/*r*. The exchange and correlation kernel for the TDDFT part was approximated by the adiabatic local density approximation (ALDA).^69^ The basis set used within the AMS/ADF database consists of Slater type orbitals (STOs) of triple-ζ polarized (TZP) quality with a frozen core up to 4p shell for Ag and 1s shell for O. Zeroth order regular approximation (ZORA)^70^ was used to account for relativistic effects.

Calculations of the spectra were carried out with the complex polarizability algorithm (polTDDFT)^50^ that extracts the spectrum from the imaginary part of the polarizability at any given photon energy, avoiding the bottleneck of Davidson diagonalization as described above. Because the applicability of polTDDFT is limited to closed-shell electronic structures, SCF calculations were first carried out to identify, if needed, the system charge leading to a closed-shell electronic configuration. The structures and charged states that reached convergence were selected for the photoabsorption spectra calculations. Both DFT structure optimizations and TDDFT photoabsorption calculations were carried out for models in a vacuum environment.

When computationally feasible (e.g., for the Ag_20_ particle), polTDDFT results were compared to those obtained with the Casida method.^62^ Photoabsorption spectra for Ag_20_ were calculated with excitation energies ranging from 0 to 5 eV, and a slightly wider range of energies was considered for Ag_140_, i.e., from 0 to 7 eV.

## 3 Results and Discussion

### 3.1 Energetics and Structure of Oxidized Ag Particles

#### 3.1.1 Adsorption of a Single O Atom on Ag_20_

We start by addressing the adsorption of a single O atom on the Ag_20_ NP. The adsorption of atomic O was evaluated on all inequivalent adsorption sites, uniquely identified by their generalized coordination number ().^71^ The  accounts for the coordination number of the site’s atoms as well as the coordination number of its first neighbors and is defined as
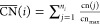
4where *n*_*i*_ is the number of nearest-neighbors of the site *i* (i.e., its coordination number), cn*(j)* is the coordination number of each neighboring metal atom *j*, and cn_max_ is the maximum coordination number of the site (e.g., 12 and 18 for top and bridge sites in an fcc structure, respectively).

The adsorption energies (calculated according to eq 1) are represented in Figure 1a as a function of their . The corresponding relaxed structures are illustrated in Figure S1a, and all *E*_ads_ and  values are collected in Table 1. We note that, for some evaluated sites, the O atom migrated to a different site upon geometry relaxation, and we therefore only consider the final states of each relaxation. The most favorable O adsorption was found on a site with  = 3.14, corresponding to a threefold hollow position near the cluster edge, with *E*_ads_ = −0.80 eV. This is followed in terms of stability by adsorption on three other sites: bridge, fourfold, and threefold hollow. We note that the fourfold site is not present in the bare particle but is formed by an adsorbate-induced reconstruction upon adsorption of O on a threefold site close to the particle corner. Adsorption over the on-top position at the cluster corner, identified by  = 1.5, is the most unstable and endothermic with respect to 1/2 of gas-phase O_2_. Based on these results, the most stable O/Ag_20_ structure ( = 3.14) was selected for the calculation of the optical properties.

**Table 1:** Adsorption Energies *E*_ads_(O) (in eV) of a Single O Atom on Different Sites of the Ag_20_ Particle (Calculated According to eq 1) and Generalized Coordination Numbers  for Each Site^a^

site		*E*_ads_(O) [eV]
top	1.5	1.143
threefold	1.73	–0.497
threefold	2.05	–0.413
bridge	2.67	–0.641
threefold	2.73	0.435
threefold	3.14	–0.800

a The corresponding relaxed structures are illustrated in Figure S1a.

#### 3.1.2 Dissociative vs Molecular Adsorption on Ag_20_

We next determined whether the adsorption of an O_2_ molecule as two O atoms (dissociative) is preferred to the molecular adsorption by comparing the stability of structures with two coadsorbed O atoms to those with an adsorbed O_2_ molecule. Dissociative O_2_ adsorption was investigated starting from the most stable O/Ag_20_ structure ( = 3.14), to which a second O atom was added in five different inequivalent sites as illustrated in Figure S2a. The corresponding adsorption energies are shown in Table 2, reported both as O_2_ adsorption energies *E*_ads_(O_2_) and as incremental O adsorption energies Δ*E*_ads_(O) with respect to the most stable O/Ag_20_ structure.

**Table 2:** Dissociative Adsorption Energies *E*_ads_(O_2_) of a Single O_2_ Molecule on Different Sites of the Ag_20_ Particle, as Illustrated in Figure S2a^a^

site	*E*_ads_(O_2_)	Δ*E*_ads_(O) [eV]
0	–1.136	–0.335
1	–1.410	–0.609
2	–1.492	–0.691
3	–1.604	–0.803
4	–1.738	–0.937

a Differential adsorption energies Δ*E*_ads_(O) corresponding to the adsorption of just the second O atom are also given.

**Figure 2:**
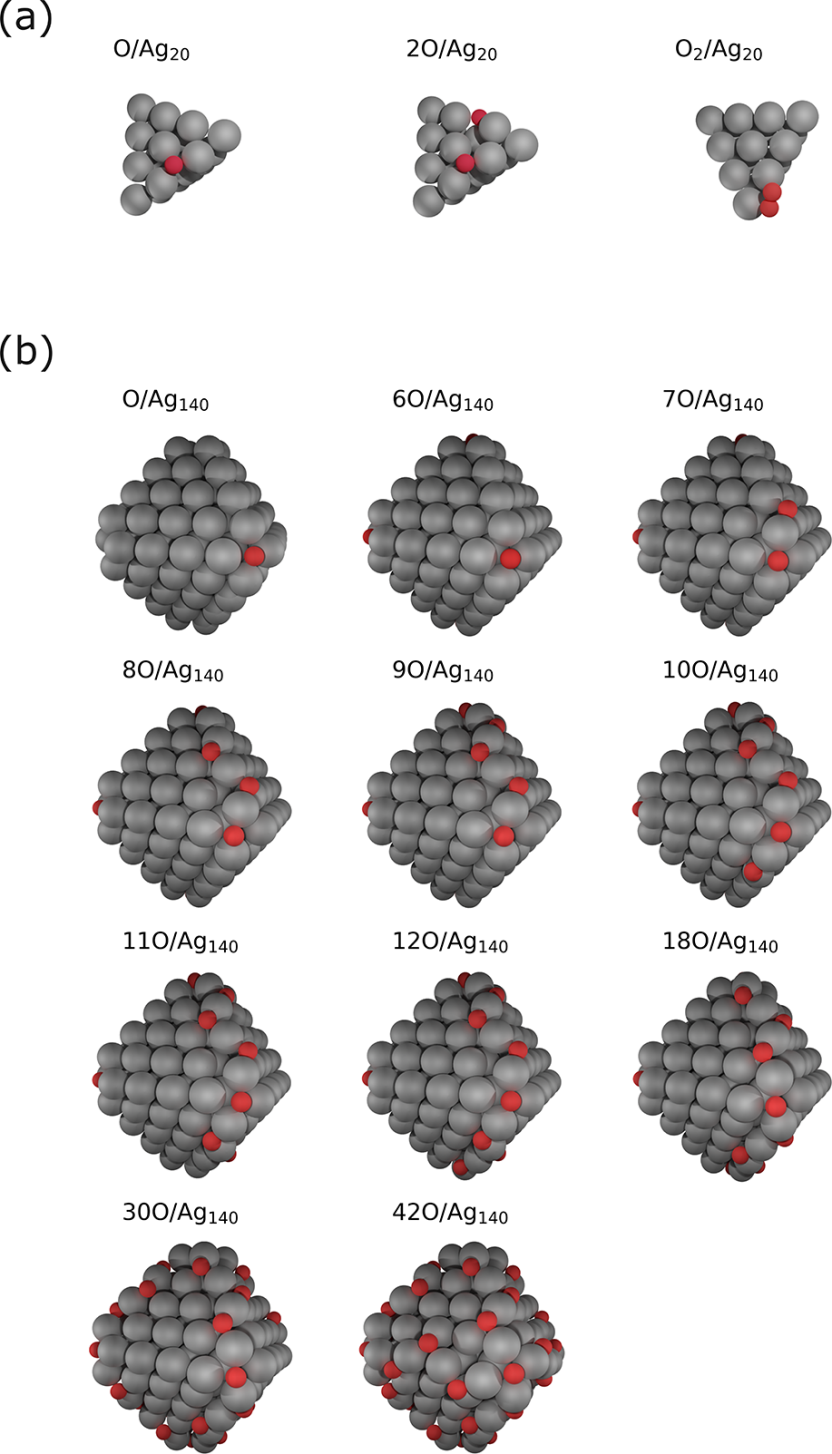
Optimized structures of O-decorated Ag_20_ (a) and Ag_140_ (b) NPs with different O coverage. The corresponding Δ*E*_ads_(O) values are collected in Tables 1, 2, and 5.

The most stable arrangement of two coadsorbed O atoms on the Ag_20_ particle corresponds to that with one oxygen atom adsorbed at a threefold hollow position near a cluster edge and the second oxygen at a threefold hollow position along the edge of a different facet (see Figure 2a and structure 4 of Figure S2a). This coadsorption leads to a slight particle reconstruction resulting in increased Ag–Ag bond distances and an *E*_ads_(O_2_) of −1.74 eV. It is worth noting that other geometries exhibiting more distant coadsorbed O atoms are less stable. This implies stabilizing lateral interactions in O-decorated Ag particles, as is shown below also for the Ag_140_ NP. The most stable 2O/Ag_20_ structure was selected for the evaluation of optical properties.

The molecular adsorption of O_2_ on Ag_20_ was examined by evaluating different on-top and bridge sites (see Figure S2b) involving coordination with a single Ag atom (η_1_-O_2_) or two Ag atoms (η_2_-O_2_), respectively. In agreement with former theoretical studies,^72^ atop binding of O_2_ is energetically favored for the considered cluster, with an *E*_ads_(O_2_) of −0.75 eV (see structure 7 of Figure S2 and Table 3). Considering the *E*_ads_(O_2_) values obtained for the dissociative adsorption (ranging from −1.14 to −1.74 eV), the latter is more energetically favorable.

**Table 3:** Molecular (Nondissociative) Adsorption Energies *E*_ads_(O_2_) of a Single O_2_ Molecule on Different Sites of the Ag_20_ Particle, as Illustrated in Figure S2b

site	*E*_ads_(O_2_) (eV)
0	–0.439
1	–0.566
2	–0.571
3	–0.615
4	–0.620
5	–0.693
6	–0.738
7	–0.745

Despite the greater stability of the structure with two coadsorbed O atoms, we also selected one O_2_/Ag_20_ structure for the evaluation of optical properties in the presence of molecularly adsorbed O_2_. Because we encountered convergence issues during SCF calculations with the AMS/ADF code for structures with the O_2_ molecule adsorbed with an on-top geometry, we finally calculated the optical properties with a slightly less stable structure in which the O_2_ molecule is adsorbed on a bridge site (*E*_ads_(O_2_) = −0.62 eV), corresponding to structure 4 in Figure S2b (also shown in Figure 2a).

#### 3.1.3 Adsorption of O Atoms on Ag_140_

Given the much greater stability discussed above of coadsorbed O atoms with respect to adsorbed O_2_ molecules on the Ag_20_ NP, we considered the adsorption only of O atoms on the Ag_140_ NP. As was done for Ag_20_, we evaluated the adsorption of a single O atom on all inequivalent sites, as defined by their generalized coordination number  (see Figure 1b, Figure S1b, and Table 4). The most stable adsorption takes place on a fourfold hollow site ( = 2.92) on the (100) facet of the NP, with an *E*_ads_(O) of −1.50 eV, followed in terms of stability by threefold hollow sites near the NP edge and corner (with  values of 4.45 and 5.05).

**Table 4:** Adsorption Energies *E*_ads_(O) (in eV) of a Single O Atom on Different Sites of the Ag_140_ Particle (Calculated According to eq 1) and Generalized Coordination Numbers  for Each Site^a^

	*E*_ads_(O) [eV]
6.95	–0.741
5.95	–0.857
6.32	–0.874
4.45	–1.098
4.45	–1.163
5.05	–1.164
2.92	–1.503

a The corresponding relaxed structures are illustrated in Figure S1b.

Based on these results, we prepared several oxidized models progressively increasing the coverage of the O atoms (see Figure 2b and Table 5). We first occupied the other five fourfold hollow sites on the {100} facets, leading to the 6O/Ag_140_ system illustrated in Figure 2b. We note that the incremental adsorption energy of these additional O atoms in the fourfold sites is progressively reduced, reaching −0.97 eV for the sixth added O atom. Notably, while searching for optimal ways of decorating the Ag_140_ particle with O, we identified a very stable arrangement of O atoms along the NP edges (Figure 2b). This energetically favorable edge oxidation involves threefold hollow sites and forms an O zigzag pattern of quasi-linear O–Ag–O motifs and angular Ag–O–Ag motifs along the NP edge. Incremental adsorption energies Δ*E*_ads_(O) of O atoms forming such motifs are as low as −1.51 eV, therefore forming significantly stronger bonds than O atoms on the same sites in the absence of other coadsorbed O atoms (which exhibit *E*_ads_(O) of ∼− 1.16 eV). This highlights the stability of these oxidized edges and indicates the presence of significantly stabilizing lateral interactions in the O-decorated Ag nanoparticles. In fact, the minimum in incremental adsorption energies (∼−1.5 eV) is reached upon completing the decoration of a whole edge, thereby contributing to the formation of two quasi-linear O–Ag–O motifs. Similar oxidized edges have been predicted and experimentally detected on stepped Pd,^73,74^ Pt,^75^ AgAu,^76,77^ Rh,^78^ and even Au^79,80^ surfaces, and similar quasi-linear O–metal–O and angular metal–O–metal motifs have recently been identified in oxidized Pt^81−83^ and Pd^84^ clusters. This suggests that the formation of such O-decorated edges is general to NPs of various transition metals and that such one-dimensional metal-oxide motifs play an important role in the chemical and physical properties of nanostructured transition metals.

**Table 5:** Incremental Adsorption Energies Δ*E*_ads_(O) of the *N*_th_ O Atom to the Corresponding (*N*– 1)O/Ag_140_ System^a^

no. of O atoms	Δ*E*_ads_(O) [eV]
1	–1.51
6	–0.97
7	–1.32
8	–1.35
9	–1.48
10	–1.20
11	–1.27
12	–1.50
18	–1.33
30	–1.33
42	–1.14

a For *n* = 18, 30, and 42, Δ*E*_ads_(O) corresponds to the average energy gain per O atom upon increasing the O coverage by 12 O atoms.

Following this stable edge-patterning scheme, we optimized the structure of particles with an increasing number of oxidized edges (see Figure 2b): 12 O atoms, for which two oxidized edges are joined by a corner but not sharing the same facet; 18 O atoms, which oxidize four edges, forming a one-dimensional oxide structure around the particle; 30 O atoms, featuring 8 oxidized edges; and 42 O atoms, for which all edges are oxidized, leading to significant structural distortions. Progressive oxidation of up to 8 edges (30 O atoms) involves similarly low incremental adsorption energies of −1.33 eV (see Table 5), and there is just a slight drop in energy gain per added O atom (to −1.14 eV) when adding up to 42 O atoms (8 oxidized edges).

The following stoichiometries were selected for evaluating the effect of increasing the O coverage on the optical properties of the Ag_140_ NP: Ag_140_, O/Ag_140_, 6O/Ag_140_, 12O/Ag_140_, and 18O/Ag_140_.

### 3.2 Optical Properties

#### 3.2.1 Photoabsorption Spectra of Bare and Oxidized Ag_20_

**Figure 3:**
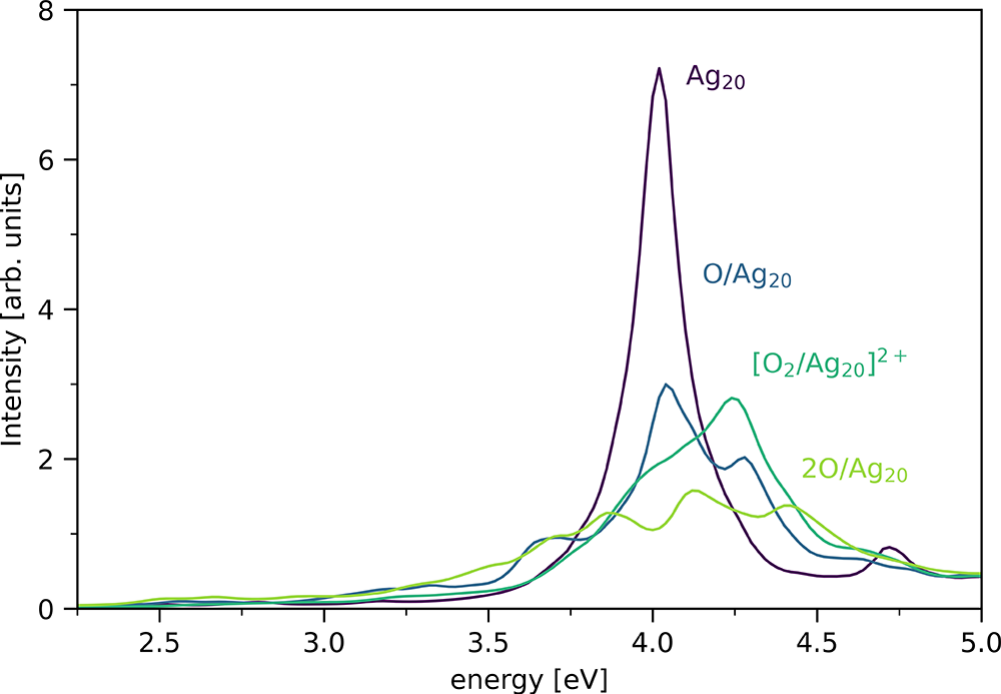
Photoabsorption spectra of Ag_20_, O/Ag_20_, [O_2_/Ag_20_]^2+^, and 2O/Ag_20_ calculated with the polTDDFT method.

The photoabsorption spectra calculated by means of the polTDDFT approach for the selected Ag_20_, O/Ag_20_, 2O/Ag_20_, and O_2_/Ag20 structures are presented in Figure 3. The spectra for Ag_20_, O/Ag_20_, and 2O/Ag_20_ were also calculated by means of the more accurate and computationally more demanding Casida method^62^ (Figure S3), which is still feasible for such small system sizes. This allows us to confirm the reliability of the polTDDFT approach, whose calculated spectra agree well with those obtained with the Casida method. We remind the reader that the polTDDFT approach only works for closed-shell systems. For structures that converge to an open-shell electronic configuration, electrons must be added or removed to achieve a closed shell electronic structure. To evaluate the effect of system modifying charge on the optical response, we have compared the spectrum of neutral O/Ag_20_ to that of [O/Ag_20_]^2–^ (see Figure S4). The peak intensity and overall shape of the neutral and negatively charged systems are very similar, and the spectra mainly slightly differ in the peak position (by ∼0.15 eV). The lower energy of peaks in the spectrum of the negatively charged system is consistent with its more populated conduction band, which lowers the energy difference between the Fermi level (or HOMO energy) and the unoccupied states that become populated upon excitation. We thus conclude that the charge of the system does not significantly affect the photoabsorption spectra and that, when required, we can reliably use charged systems to model the optical properties of the different structures.

The spectrum of the bare Ag_20_ cluster is characterized by the presence of a strong and sharp peak centered at 4.02 eV. The energy position of the peak and its shape are characteristic of a plasmon resonance.^18^ The peak at 4.02 eV is in good agreement with previous TDDFT results^20,85^ and with experimentally measured spectra (albeit slightly blue-shifted).^86^ In turn, the spectrum of O/Ag_20_ is characterized by a splitting of the signal into various less intense peaks and an overall decrease of intensity. The splitting of the signal is mainly due to the loss of symmetry introduced upon adsorption of oxygen^25^ and, to some extent, to the O-induced Ag_20_ reconstruction. Furthermore, the decrease in intensity can be ascribed to an electronic effect emerging from the oxidation of the Ag atoms in the vicinity of the adsorbed oxygen atom. To validate this hypothesis, the photoabsorption spectrum of the distorted Ag_20_ structure was computed by removing the O atom from the O/Ag_20_ structure but keeping the distorted geometry fixed. The resulting spectrum is compared to those of O/Ag_20_ and the undistorted Ag_20_ particle in Figure S5, where the similar signal splitting of distorted Ag_20_ and O/Ag_20_ confirms that such spitting is due to the structural distortion. In turn, the intensity of each of the two peaks for the distorted Ag_20_ is nearly half of the intensity of the original peak for the undistorted particle. Thus, the intensity seems to be affected not by the loss of crystallinity but by the electronic effect of bonding to O.

These first results indicate that the presence of oxygen has a dramatic effect on the optical properties of the Ag_20_ particle, essentially quenching the plasmonic response. This effect is even more pronounced when increasing the number of adsorbed oxygen atoms; for 2O/Ag_20_ (see Figure 3 and Figure S3c), the presence of two O atoms is enough to induce the complete loss of the plasmonic peak. In turn, the weaker interaction with molecular O_2_ than with two coadsorbed O atoms leads to a weaker disruption of the plasmonic peak. The photoabsorption spectrum of the O_2_/Ag_20_ system is therefore similar to that of the O/Ag_20_. This indicates that the dissociation event of an O_2_ molecule on Ag_20_ could be detected by monitoring the photoabsorption spectrum, although photoexcitation can also facilitate such dissociation.^88−91^

**Figure 4:**
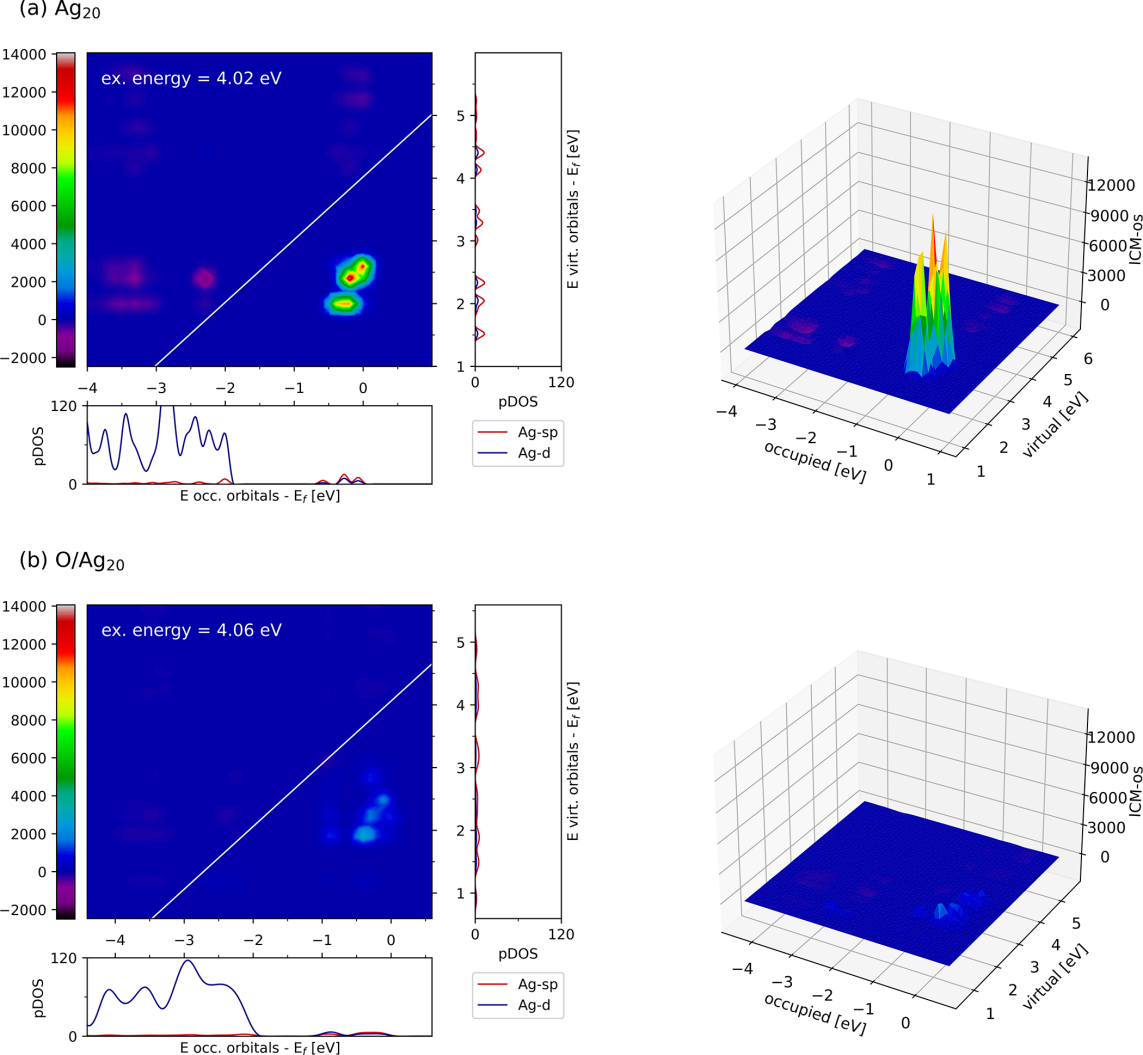
ICM-OS of Ag_20_ (a) and O/Ag_20_ (b) for excitation energies of 4.02 and 4.06 eV, respectively. Orbital energies are shifted with respect to HOMO (i.e., the Fermi level). For each system, the DOS projected on the *sp* or *d* states of Ag are plotted along the axes of the ICM-OS. 3D versions of the plots are also included (right-hand panels) for clarity.

To better assess the effect of adsorbed O on the plasmonic response, the main peak in the photoabsorption spectra of Ag_20_ and O/Ag_20_ was analyzed by means of the Individual Component Map of the Oscillator Strength (ICM-OS).^92,93^ This tool allows analyzing a specific adsorption peak in terms of the contributions of pairs of occupied and virtual orbitals. It thus associates a specific orbital character with an electronic transition. The ICM-OSs for Ag_20_ and O/Ag_20_ are shown in Figure 4 using both 2D and 3D representations. The orbital energies in these plots are expressed with respect to the HOMO energy (i.e., the Fermi level) of each system, and the diagonal white line in the 2D plots displays the difference in orbital energies corresponding to the energy of the exciting photon. Spots on the diagonal (if any) would therefore correspond to excited configurations with the same energy of the photon, whereas off-diagonal spots indicate a collective behavior typical of plasmons in which the strong coupling between excitations modifies their energies. In addition to the weight of each pair of orbitals on the oscillator strength, ICM-OS also takes into account dipole contributions, which might result in constructive or destructive interference among excited configurations. In fact, a destructive dipole contribution is apparent in the regions between −2 and −4 eV of the occupied orbitals and from 2 to 3 eV of the unoccupied orbitals.

The presence of some off-diagonal spots in the ICM-OS plots of Ag_20_ is characteristic of a plasmonic system because it emerges from the coupling from different single excited configurations. The ICM-OS plots comprise only a few spots because the collective nature of the excitations is limited by the small particle size. In the case of O/Ag_20_, the ICM-OS plots show off-diagonal spots at the same energetic positions as the spots of the bare cluster, but the intensity is considerably lower, whereas no such spots are seen for the 2O/Ag_20_ system (not shown). This evolution of the off-diagonal spot intensity is fully consistent with the outcomes of the photoabsorption spectra.

To pinpoint the character of the orbitals involved in the plasmonic excitation, the projected density of states (pDOS) of the occupied and unoccupied states of the (neutral) Ag_20_ and O/Ag_20_ systems is plotted along the axes of the corresponding ICM-OS plots in Figure 4. The spots within the ICM-OS plots correspond to DOS regions dominated by *sp* states of silver (with small contributions from *d* states) for both the occupied (at around −1 to 0 eV) and unoccupied (at around 2 to 3 eV) states. Thus, the single particle transitions that contribute to the collective behavior all occur within the *sp* band and close to the Fermi level, in agreement with previous work.^20,26,94,95^ We note that both the *sp* and *d* pDOSs near the Fermi level for Ag_20_ do not resemble much of a band and instead have a more discrete form, which is consistent with the discrete nature of the electronic states for clusters of such size. Most importantly, the *sp* pDOS is smoothed out upon oxygen adsorption due to the hybridization of these states with those of the O atom. In fact, the *sp* pDOS between −1 and 0 eV below the Fermi level is significantly flattened for the O/Ag_20_ and even more so for 2O/Ag_20_ systems (see Figure 4 and Figure S6). This indicates that the plasmon quenching is at least partially due to such hybridization, which lowers the density of states responsible for the coupled single particle excitations. The bonding to the O atoms also effectively increases the atomic charge of the involved Ag atoms, giving rise to Ag^δ+^ cations with fewer 5s electrons and therefore less ability to support the plasmon resonance. The quenching of the plasmon upon O adsorption agrees with the results by Schira and Rabilloud for Ag_8_, Ag_20_, and Ag_38_ particles decorated with up to three O atoms.^26^ This behavior is also analogous to the reduction of plasmon intensity reported for Au clusters upon oxidation, which also makes 6s electrons less available.^96^

To further confirm the ionic character of the Ag atoms in the presence of O, we evaluated the evolution of the Ag Bader charges^97^ upon oxidation. Bader charges Δ*q* were computed as differences between the atomic charges that arise upon oxygen adsorption and the charges of the same Ag atoms in the bare NP. In this way, one gets rid of effects related to the larger volumes assigned to surface atoms, which can spuriously lead to negatively charged surface atoms and positively charged bulk-like atoms. The Bader charges shown in Figure S7 confirm that Ag atoms in contact with adsorbed oxygen are rather oxidized, with charges of up to +0.3 |**e**|. The total charge of each O atom, ∼−0.9 |**e**|, indicates the decreased number of electrons able to contribute to the plasmon. It is worth noting that whereas the plasmon is only slightly affected by cluster charge, it is strongly affected by the formation of the Ag^δ+^ cations when oxidation occurs. The behavior is different because in the former, the total charge is redistributed uniformly within the cluster, producing a “rigid” shift of the energy of the molecular orbitals. On the other hand, when a strong Ag^δ+^–O^δ^ dipole is formed, its anisotropic potential prevents the electrons of the remaining Ag atoms to move as freely as in the absence of the dipole.

**Figure 5:**
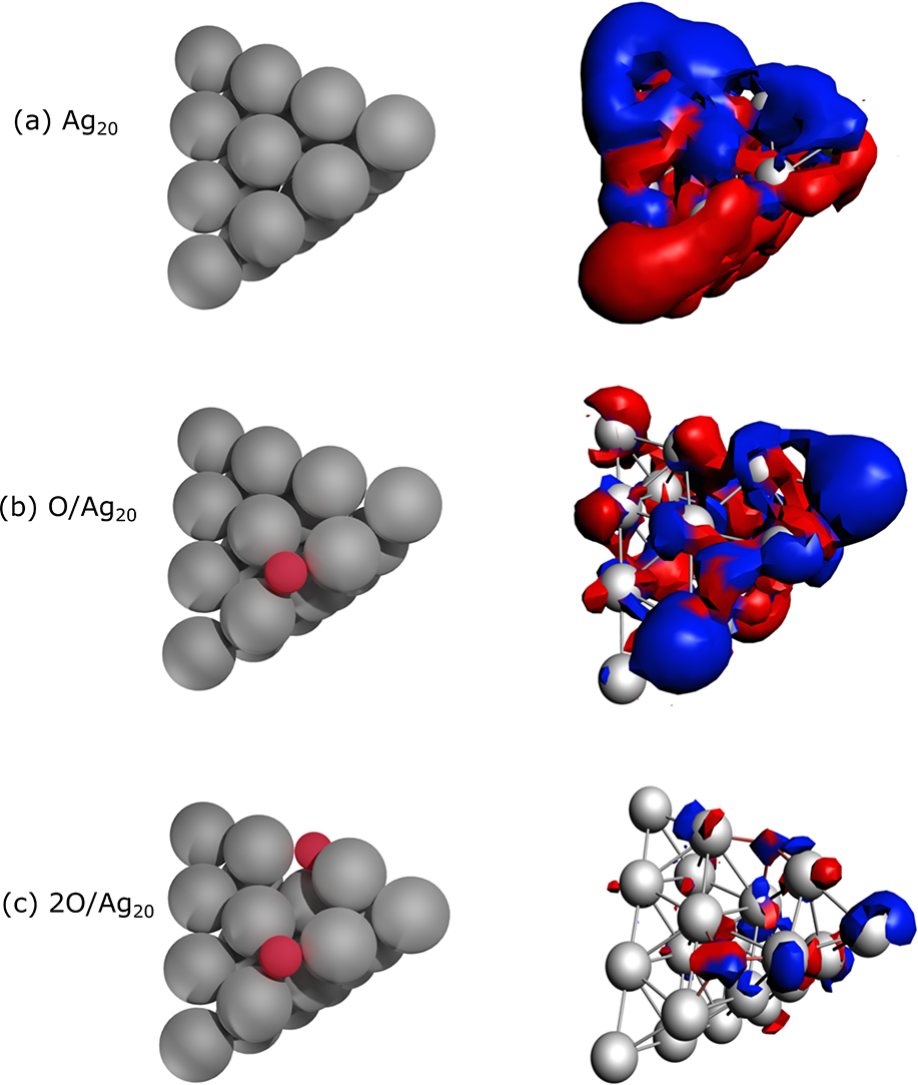
Induced density at the plasmonic resonance for (a) Ag_20_, (b) O/Ag_20_, and (c) 2O/Ag_20_. Blue and red indicate the positive and negative contributions, respectively, of the induced charge density. Density values of 0.3 **e**/Å^3^ were used for defining the isosurfaces. Only the component along the *y* axis is shown.

Plots of the density induced by the plasmonic resonance shown in Figure 5 illustrate how the charge density is redistributed during the transition. They were calculated at the energy corresponding to the maximum of the oscillator strength in the corresponding photoabsorption spectra. Blue and red indicate the positive and negative contributions, respectively, of the induced charge density. For clarity, only one component of the latter (the one along the *y* axis) is shown. The induced density for the bare Ag_20_ particle shown in Figure 5a exhibits a dipolar shape, which is expected given the plasmonic nature of such cluster.^19^ The induced density of the O/Ag_20_ system (Figure 5b) is in turn significantly altered, with Ag atoms in the vicinity of the O atoms displaying a different (blue) phase and without a distinguishable dipole. Because of the hybridization and charge depletion from Ag atoms upon bonding to O, the charge density and its contributions are reduced. This is also the case for the 2O/Ag_20_ system (Figure 5c), where the induced charge density of the bare Ag_20_ particle nearly vanished, further demonstrating the complete suppression of the plasmon.

#### 3.2.2 Photoabsorption Spectra of Bare and Oxidized Ag_140_

**Figure 6:**
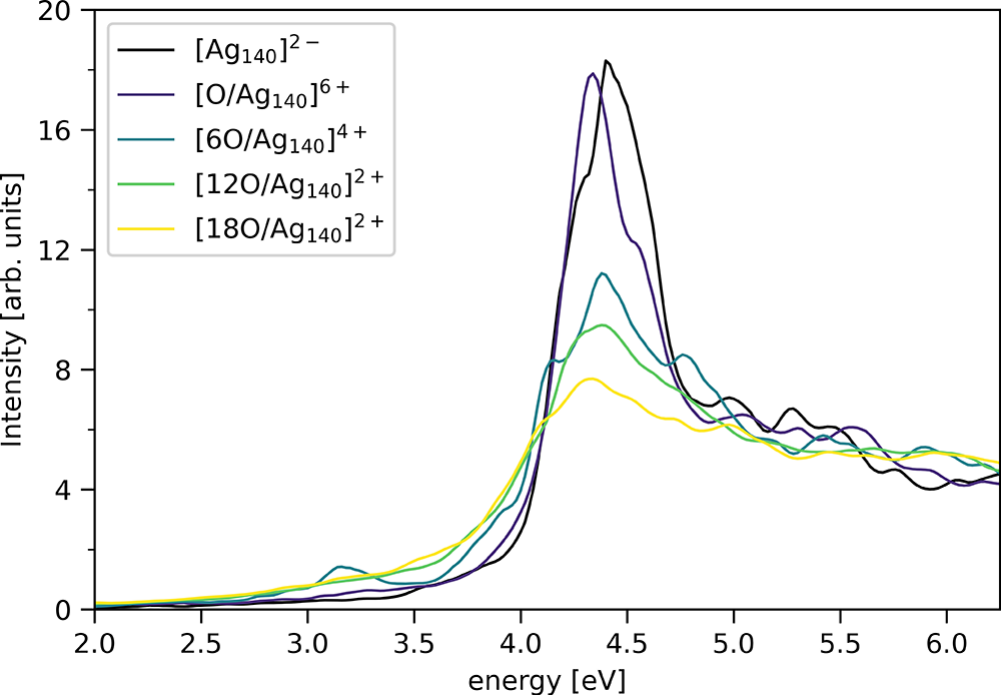
Photoabsorption spectra of the [Ag_140_]^2–^, [O/Ag_140_]^6+^, [6O/Ag_140_]^4+^, [12O/Ag_140_]^2+^, and [18O/Ag_140_]^2+^ systems calculated with the polTDDFT method.

To evaluate the effect of oxidation on the optical properties of larger Ag NPs, we calculated the photoabsorption spectra (at the polTDDFT level only), ICM-OS plots, pDOS, Bader charges, and induced densities of selected Ag_140_ structures with an increasing coverage of O (as described in Section 3.1.3). In particular, we have evaluated Ag_140_, O/Ag_140_, 6O/Ag_140_, 12O/Ag_140_, and 18O/Ag_140_, which were found to converge to closed-shell electronic configurations for system charges of *q* = −2, +6, +4, +2, and +2, respectively. The polTDDFT spectra are reported in Figure 6. The spectrum for the bare [Ag_140_]^2–^ system is characterized by a strong and sharp absorption peak around 4.4 eV followed by a region with only minor features. The energy position of the peak and its shape indicate its plasmonic resonance character. The main peak of the Ag_140_ particle lies at energies ∼0.3 eV higher than that calculated for Ag_20_ (see Figure S8 for a direct comparison). This is consistent with the evolution toward higher peak energies with increase in size measured^98^ and calculated^20,86,99^ for Ag particles in this size range and in contrast to the decrease in peak energy with particle size measured^18^ and calculated^21^ for larger (2 to 8 nm) particles. We note that particle shape also influences the optical properties,^99^ and therefore, the different shapes of the Ag_20_ (tetrahedral) and Ag_140_ (octahedral) NPs could be partially responsible for the calculated resonance energies. Most importantly, the increase of the coverage of the O atoms progressively affects the optical spectrum, leading to a dramatically reduced response for the [18O/Ag_140_]^2+^ system with respect to the bare NP. We still observe a peak at nearly the same energy (yet slightly red-shifted), but it has been strongly weakened and broadened. As was calculated for Ag_20_, the plasmonic resonance is, therefore, proportionally quenched as the number of adsorbed oxygen atoms increases.

**Figure 7:**
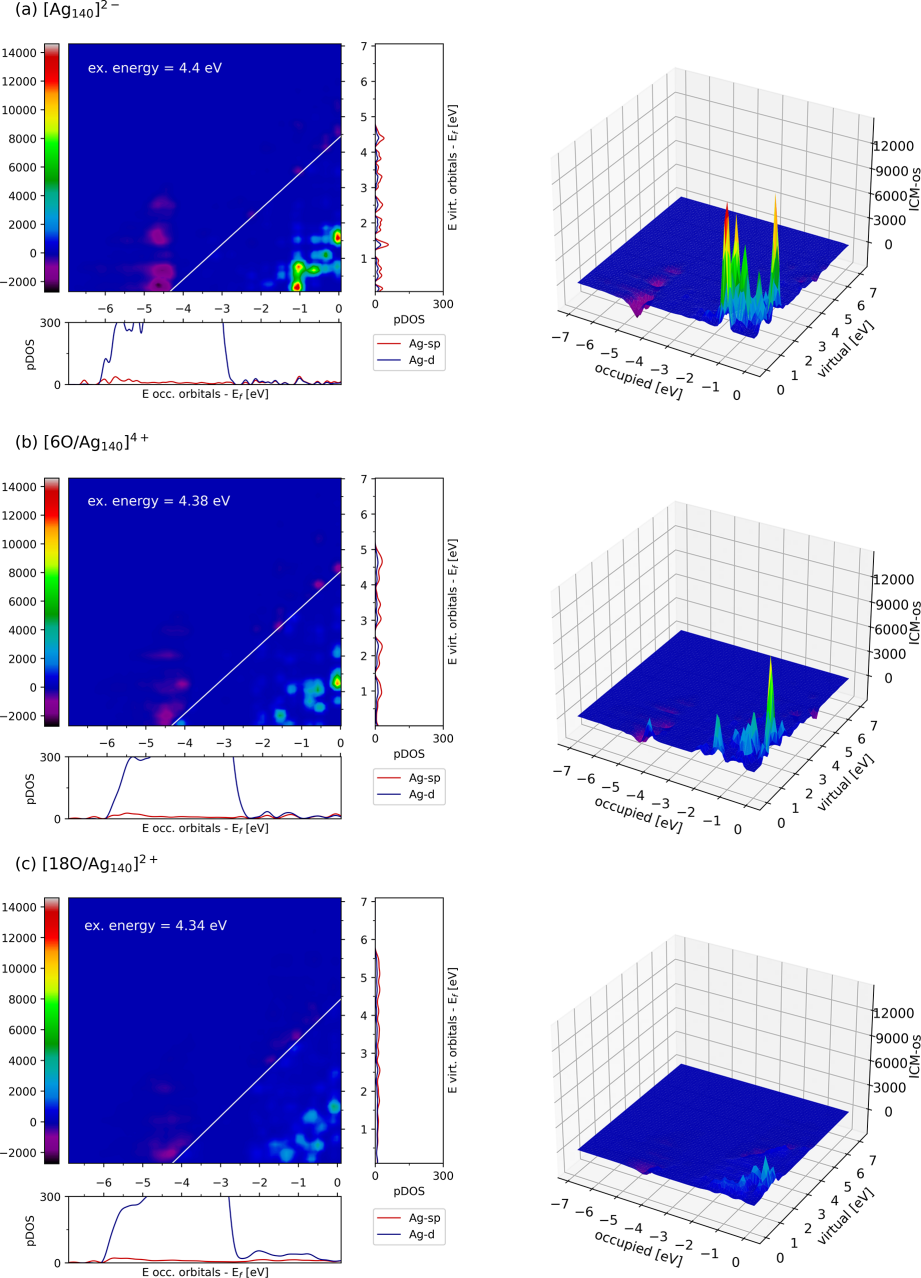
ICM-OS of Ag_140_, 6O/Ag_140_, and 18O/Ag_140_ for excitation energies of 4.4, 4.38, and 4.34 eV, respectively. Orbital energies are shifted with respect to HOMO (i.e., the Fermi level). For each system, the DOSs projected on the *sp* or *d* states of Ag are plotted along the axes of the ICM-OS. 3D versions of the plots are also included (right-hand panels) for clarity.

To better assess the effect of O coverage increase, we again focus on the ICM-OS of the main peak in the photoabsorption spectra for different stoichiometries.^92,93^ The ICM-OS analysis for [Ag_140_]^2–^, [6O/Ag_140_]^4+^, and [18O/Ag_140_]^2+^ is shown in Figure 7, using both 2D and 3D representations to better illustrate the presence and reduction of individual contributions from pairs of occupied and unoccupied orbitals. As we saw for the bare Ag_20_ particle, the ICM-OS of [Ag_140_]^2–^ is typically plasmonic, with many off-diagonal spots as indication of a collective behavior. The most intense spots in the ICM-OS plots gather around −1 eV in the occupied orbital axis and belong to single excitations occurring within the *sp* band. In turn, for this particle size, we see an even more significant destructive dipole contribution than for Ag_20_ in the energy region dominated by the *d* band of silver between −4 and −5 eV of the occupied orbitals and from 0 to 2 eV of the unoccupied orbitals. Electrons in the 5s band constructively contribute to the plasmon resonance, whereas those in the 4d band slightly reduce the plasmon intensity. The latter is a well-known screening effect by *d* electrons,^100,101^ whereby the *d*-electron transitions result in counter-polarized dipoles with respect to those induced by the *sp* transitions.^96^ The ICM-OS plots for [6O/Ag_140_]^4+^ and [18/Ag_140_]^2+^ show a gradual reduction in the intensity of the main orbital contributions to the plasmon resonance, which explains why the plasmon is much weaker than that of the bare Ag_140_ NP. Tracing these effects back to the electronic structure, we see some differences in the pDOS for the neutral Ag_140_, 6O/Ag_140_, 18O/Ag_140_, and 42O/Ag_140_ NP with respect to the Ag_20_ case (see Figure 7 and Figure S9). For the bare Ag_140_ particle, the energy regions with the largest contributions to the plasmon resonance also contain localized states. However, in contrast to the predominant *sp* character of these states for the Ag_20_ particle, the larger Ag_140_ NP exhibits almost equal contributions of *sp* and *d* states in the DOS near the Fermi level. However, the *d* states provide just a minor quenching effect. These states are delocalized upon hybridization with orbitals of the adsorbed O atoms, leading to a higher density of *d* states than *sp* states in the energy region relevant to the plasmon resonance. Adsorption of O therefore significantly affects the relative density of *sp* and *d* states, which for the 42O/Ag_140_ system results in a sizable contribution of O states hybridized with Ag 4*d* states populating the region between −2 eV and the Fermi level.

**Figure 8:**
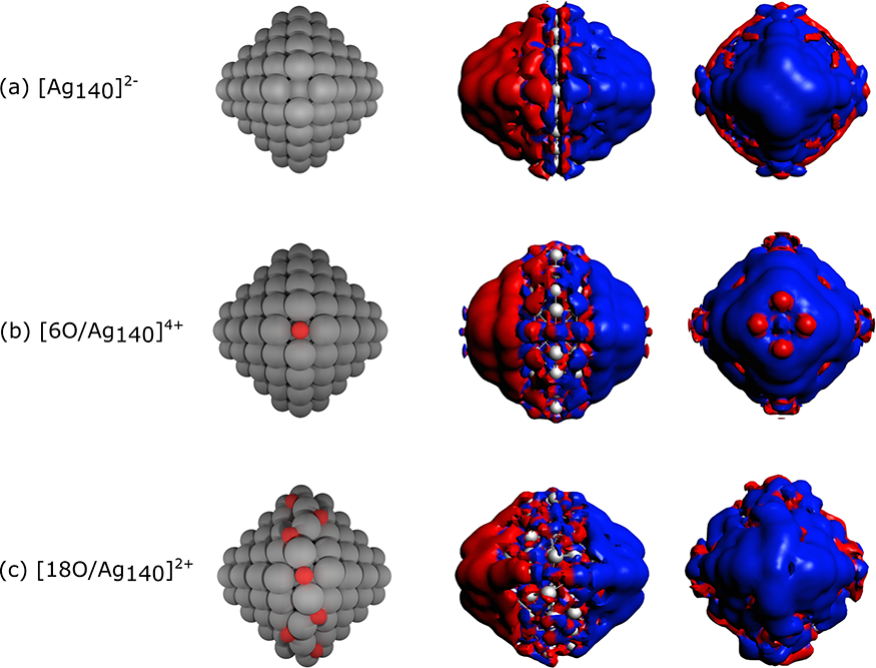
Induced density at the plasmonic resonance for [Ag_140_]^2–^, [6O/Ag_140_]^4+^, and [18O/Ag_140_]^2+^_._ Blue and red indicate the positive and negative contributions, respectively, of the induced charge density. The *x* (middle column) and *y* (right column) components of the induced density are shown, for which density isovalues of 0.05 and 0.03 **e**/Å^3^, respectively, were used for defining the isosurfaces.

The induced density plots for [Ag_140_]^2–^, [6O/Ag_140_]^4+^, and [18O/Ag_140_]^2+^ are reported in Figure 8. For the bare cluster, the typical plasmonic dipolar shape of the induced density is apparent. For [6O/Ag_140_]^4+^ and [18O/Ag_140_]^2+^, the induced density still retains a dipolar shape, but there is a central belt of silver atoms that do not participate in the induced density. These are Ag atoms in the vicinity of oxygen, providing a real-space illustration of how oxidation prevents their contribution to plasmon resonance. The *z* component of the [6O/Ag_140_]^4+^ induced density also exhibits a peculiar feature. The Ag atoms located at the {100} facet of the Ag NP from the fourfold hollow site that binds O have a different phase than the neighboring Ag atoms, which indicates their destructive contribution to the induced density. It is worth pointing out that unlike the 2O/Ag_20_ system, the adsorption of 18 O atoms does not completely suppress the plasmon resonance, as it exhibits a less extensive but still clearly present dipolar shape of the induced density.

The Bader charge analysis for 6O/Ag_140_, 18O/Ag_140_, 30O/Ag_140_, and 42O/Ag_140_ (Figure S10) reveals the progressive formation of additional cationic Ag^δ+^ centers upon increasing the O coverage. The adsorption of an O atom at each of the six fourfold hollow sites of the NP produces a localized electron depletion of the Ag atoms forming these adsorption sites. Contact to just one atom leads to atomic charges of ∼0.3 |**e**|, similar to the oxidation of the Ag_20_ particle. The oxidation is more pronounced for Ag atoms involved in the zigzag oxidation along the edges of the Ag particle (i.e., for the systems with 12, 18, 30, and 42 O atoms). Because every Ag atom in an oxidized edge is bound to 2 O atoms, the corresponding Ag Bader charges increase to ∼0.6 |**e**|. We note that despite the significant effect of edge oxidation on the global plasmonic character, the effect on the Ag atomic charges is still rather local. Ag atoms in bulk positions or surface positions distant from the oxidized edges essentially retain the neutral character of the bare particle.

## 4 Conclusions

In this work, we evaluated the effect of oxygen adsorption on the optical properties of Ag_20_ and Ag_140_ NPs by DFT and TDDFT calculations, focusing on the underlying changes in electronic structure upon increasing the oxygen coverage. Both Ag_20_ and Ag_140_ particles exhibit a clear plasmonic behavior, evidenced by the presence of intense and sharp photoabsorption peaks that result from collective and coupled excitations from and to states near the Fermi level. The unoccupied and occupied states contributing to such excitations are rather localized and have a mainly *sp* character for the Ag_20_ particle and a more mixed *sp* and *d* character for the Ag_140_ NP. Most importantly, these states are hybridized with the 2*sp* states of O upon Ag–O bond formation, resulting in a significant delocalization. Such delocalization, together with the oxidation of Ag atoms to Ag^δ+^ cations and the adsorbate-induced reconstructions of the Ag particles, leads to the progressive quenching of the plasmon resonance. Two adsorbed O atoms are enough to completely destroy the plasmonic behavior of the Ag_20_ particle, whereas for the larger Ag_140_ particle, 18 O atoms do not yet completely suppress the plasmon resonance.

We note that the dissociative adsorption of O_2_ on Ag_20_ is thermodynamically preferred over the molecular adsorption. Because the weaker interaction of the molecularly adsorbed O_2_ also has a smaller effect on the optical response, we suggest that the O_2_ dissociation events can be reliably measured by monitoring the plasmon intensity. In addition, we have identified particularly stable O/Ag NPs characterized by strongly oxidized edges with a zigzag O–Ag–O–Ag-O pattern. The formation of such zigzag motifs demonstrates the presence of strongly attractive lateral interactions between O atoms adsorbed on Ag NPs and is highly relevant in Ag-based catalysts under atmospheric or oxidizing reaction conditions. Our understanding of the catalytic properties of such systems may therefore be incomplete without considering the contributions from these likely prevalent edge motifs.

Our work has implications for plasmon-mediated applications in, e.g., photodetection,^14^ photocatalysis,^15^ solar energy conversion,^16^ photoelectrocatalysis,^102^ and related fields^17^ because the quenching of the plasmon upon particle oxidation would potentially limit their applicability. This is the case particularly for photocatalytic applications in which plasmonic excitations are used to dissociate adsorbed molecules.^89−92^ The resulting increase in the coverage of dissociated species (especially in the case of O_2_) is likely to quench the plasmon resonance and thereby suppress the plasmon-mediated catalytic mechanism. A similar scenario should be expected for Ag particles in reactive solvents or on supports, where the (typically blue) shifts in excitation energies induced by complex dielectric environments^100^ would be combined with the strong hybridization of the Ag *sp* states. On the other hand, regulating the oxidation state of metal NPs (e.g., by O_2_ exposure or interactions with supports^103−106^) should offer opportunities to control the intensity of plasmonic excitations, expanding the size, shape, and composition toolbox of parameters used to tune the optical properties of systems based on plasmonic nanoparticles.

## Data Availability

The outputs from the calculations underlying this study are openly available in ioChem-BD repository at 10.19061/iochem-bd-6-296.^107^

## Supporting Information Available

Additional adsorption geometries, photoabsoprtion calculations with the Casida method, charge and geometry distortion effects on the photoabsorption spectrum, and Bader charge analyses (PDF)
