# Supplementary material for: Effects of Oxygen Adsorption on the Optical Properties of Ag Nanoparticles
Source: J Phys Chem A. 2023 Dec 1;127(49):10412–24. doi: 10.1021/acs.jpca.3c05801 (PMC10726366; doi:10.1021/acs.jpca.3c05801)
Supplement: Supplementary file 1 — jp3c05801_si_001.pdf [file jp3c05801_si_001.pdf]

Supporting Information for:

Effects of Oxygen Adsorption on the Optical Properties of Ag Nanoparticles

Elena Zerbato[1], Riccardo Farris[2], Giovanna Fronzoni[1], Konstantin M. Neyman[2,3], Mauro Stener[1*], and Albert Bruix[2*]

[1] Dipartimento di Scienze Chimiche e Farmaceutiche, Università di Trieste, Via L. Giorgieri 1, 34127 Trieste, Italy.

[2] Departament de Ciència del Materials i Química Física & Institut de Química Teòrica i Computacional, Universitat de Barcelona, 08028 Barcelona, Spain

[3] ICREA (Institució Catalana de Recerca i Estudis Avançats), 08010 Barcelona, Spain

*Corresponding authors: stener@units.it, abruix@ub.edu

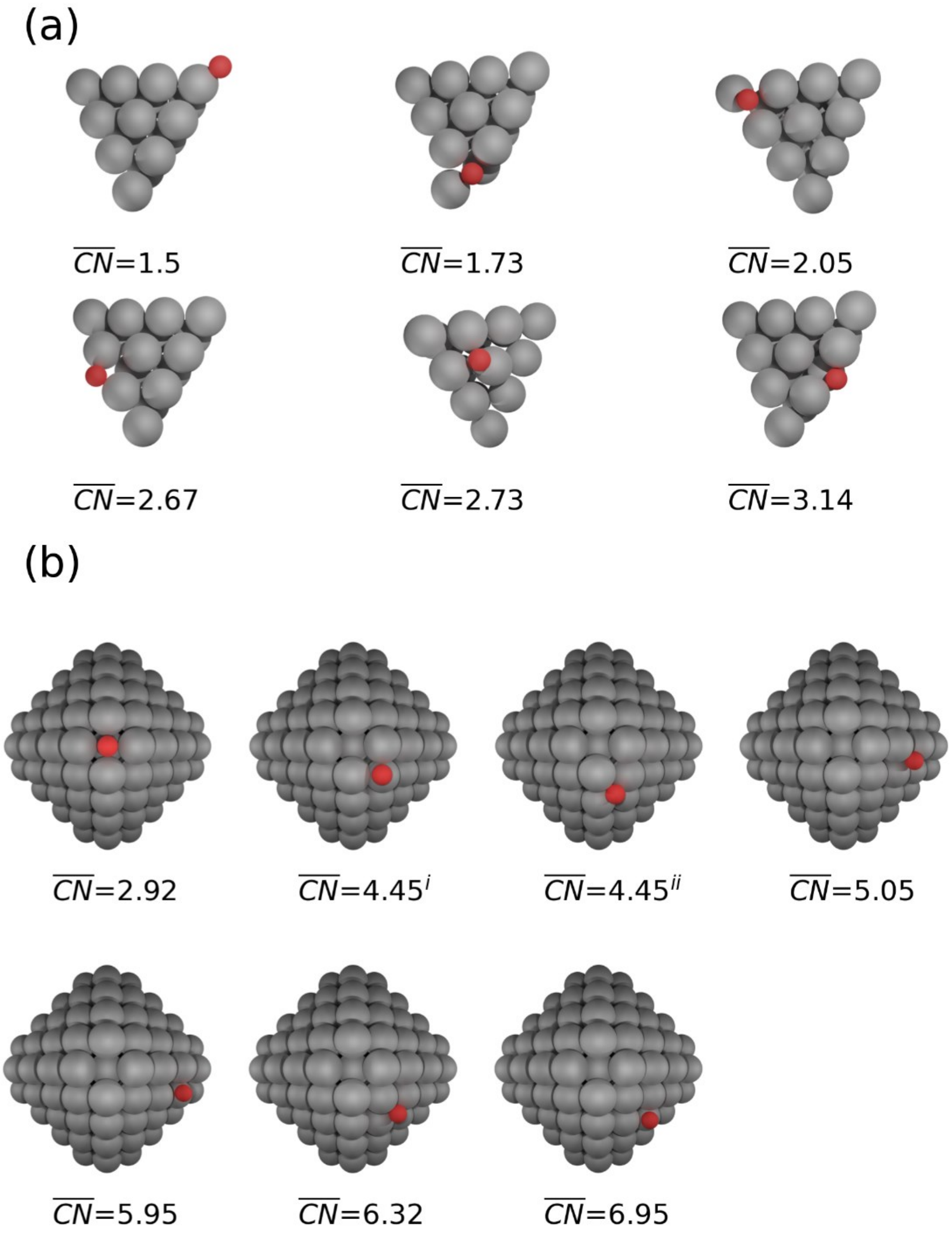

Figure S1: Optimized structures of Ag_{20} (a) and Ag_{140} (b) particles with a single O atom adsorbed at different positions defined by the site's generalized coordination number $\overline{CN}$. The corresponding ΔE_{ads}(O) values are collected in Tables 1 and 3 and plotted in Figure 1 of the article.

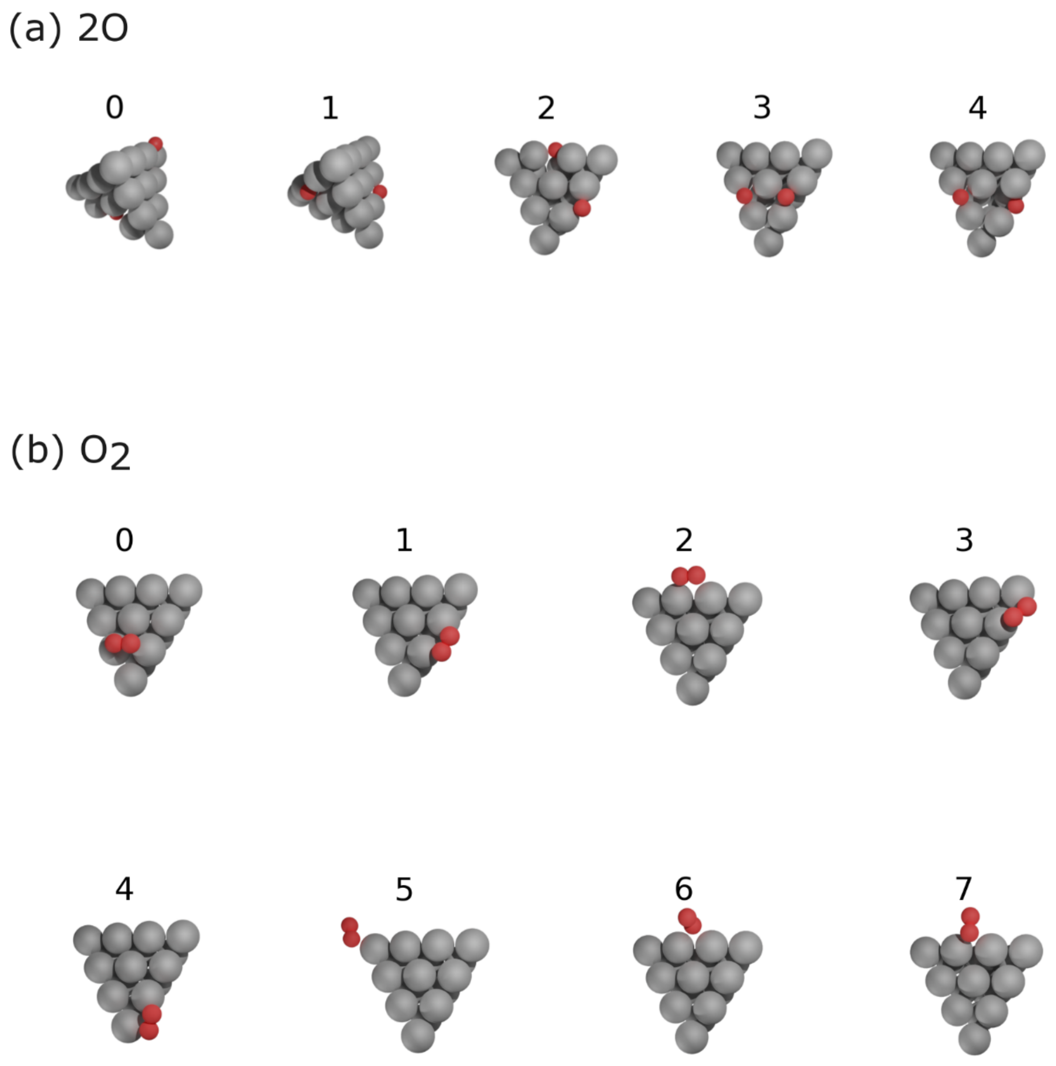

Figure S2: Evaluated structures for 2 co-adsorbed O atoms (a) and adsorbed O_2 molecule (b) on different sites of the Ag_{20} particle. The corresponding adsorption energies are shown in Tables 2 and 3.

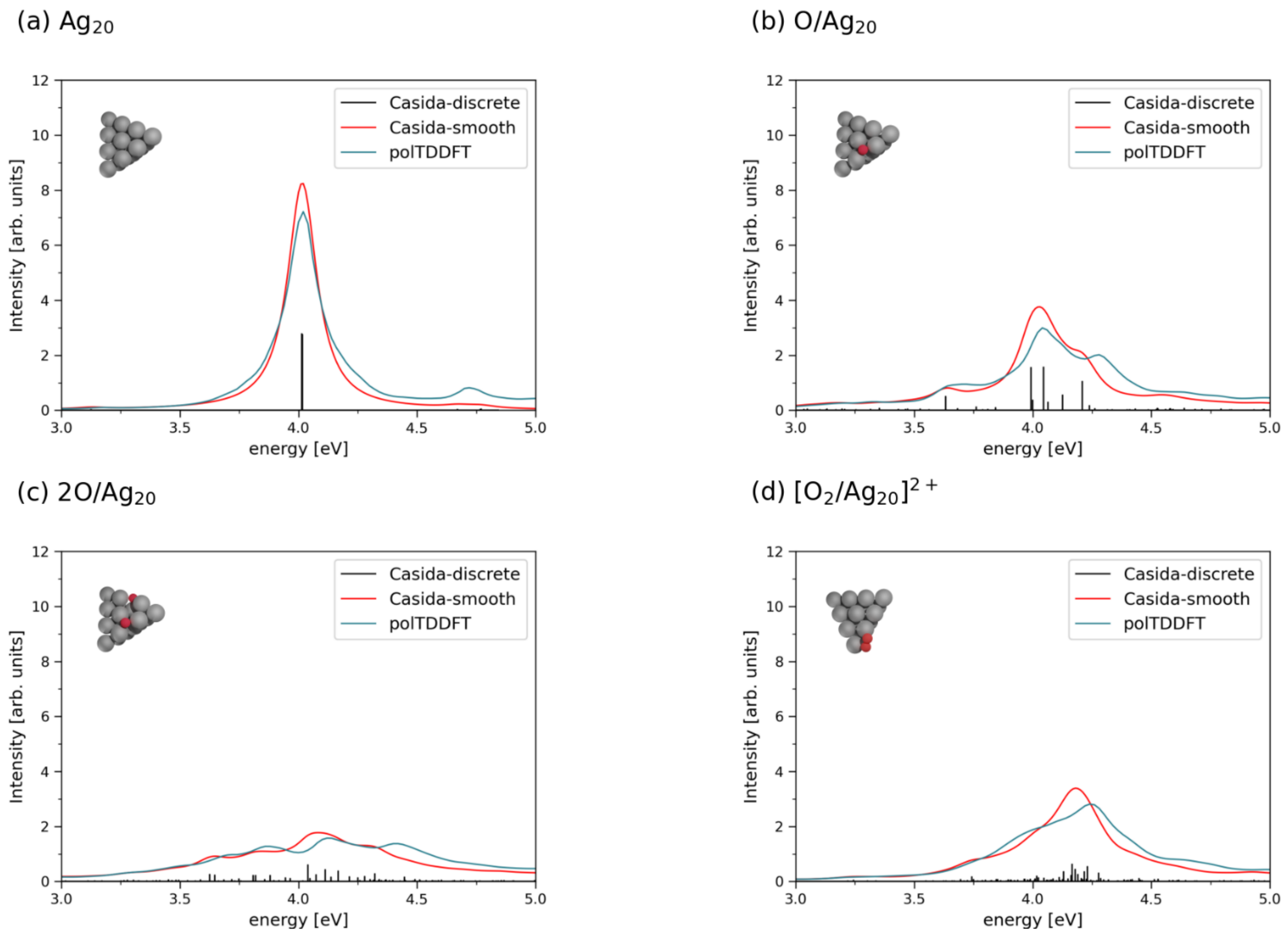

Figure S3: Photoabsorption spectra calculated by means of both the Casida and polTDDFT approaches for (a) Ag_{20}, (b) O/Ag_{20}, (c) $2O/Ag_{20}$, and (d) O_2/Ag_{20}. Casida-discrete (black vertical lines) correspond to the discrete values obtained by the Casida approach, and Casida-smooth (red lines) corresponds to the spectra resulting from these discrete values smoothened with Lorentzian functions.

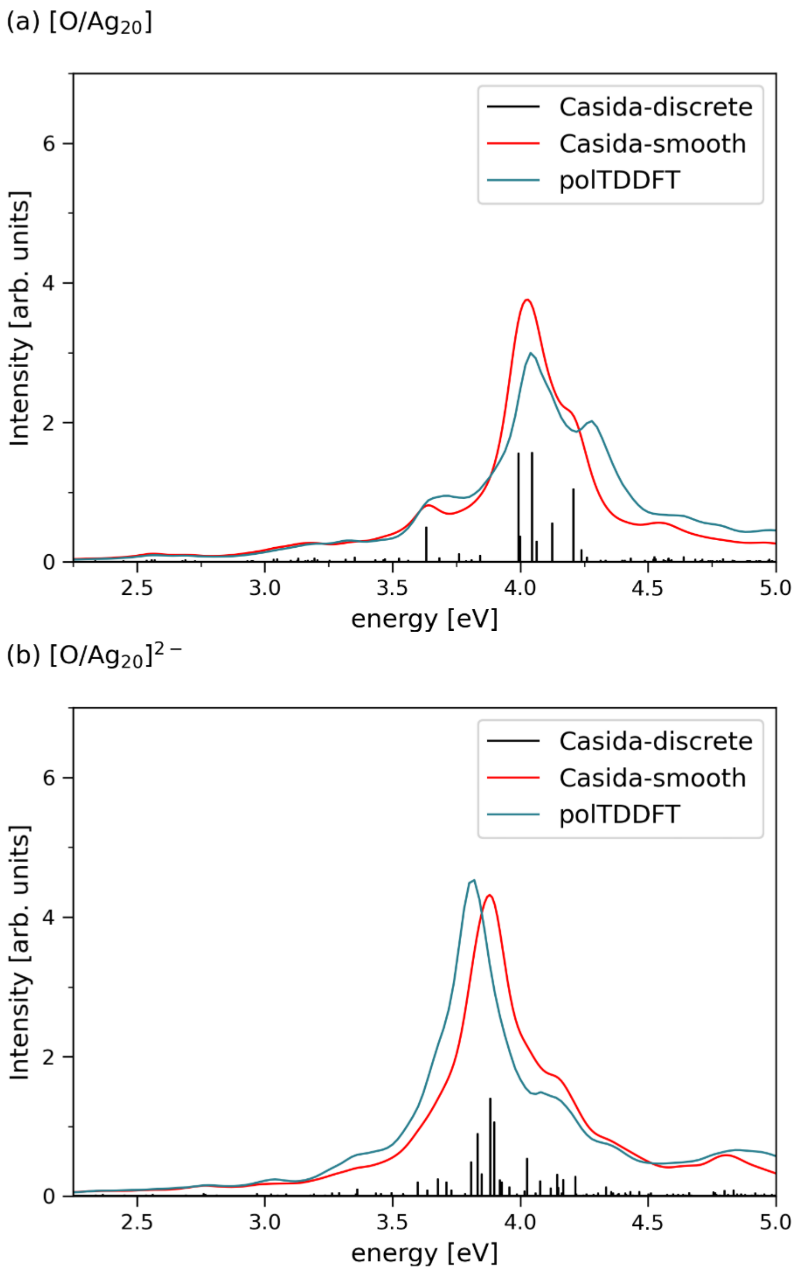

Figure S4: Photoabsorption spectra for the (a) neutral [O/Ag_{20}] and (b) negatively charged [O/Ag_{20}]$^{2-}$ systems calculated with both the Casida and polTDDFT approaches. Casida-discrete (black vertical lines) correspond to the discrete values obtained by the Casida approach, and Casida-smooth (red lines) corresponds to the spectra resulting from these discrete values smoothened with Lorentzian functions.

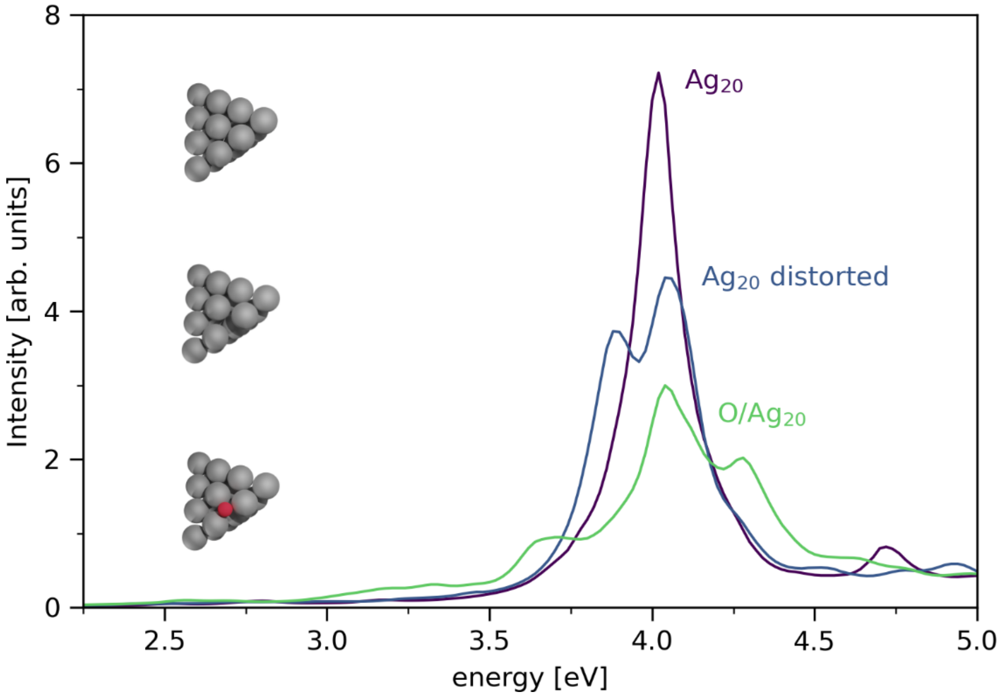

Figure S5: Photoabsorption spectra for the Ag_{20}, adsorbate-distorted Ag_{20} and O/Ag_{20} systems calculated with the polTDDFT approach.

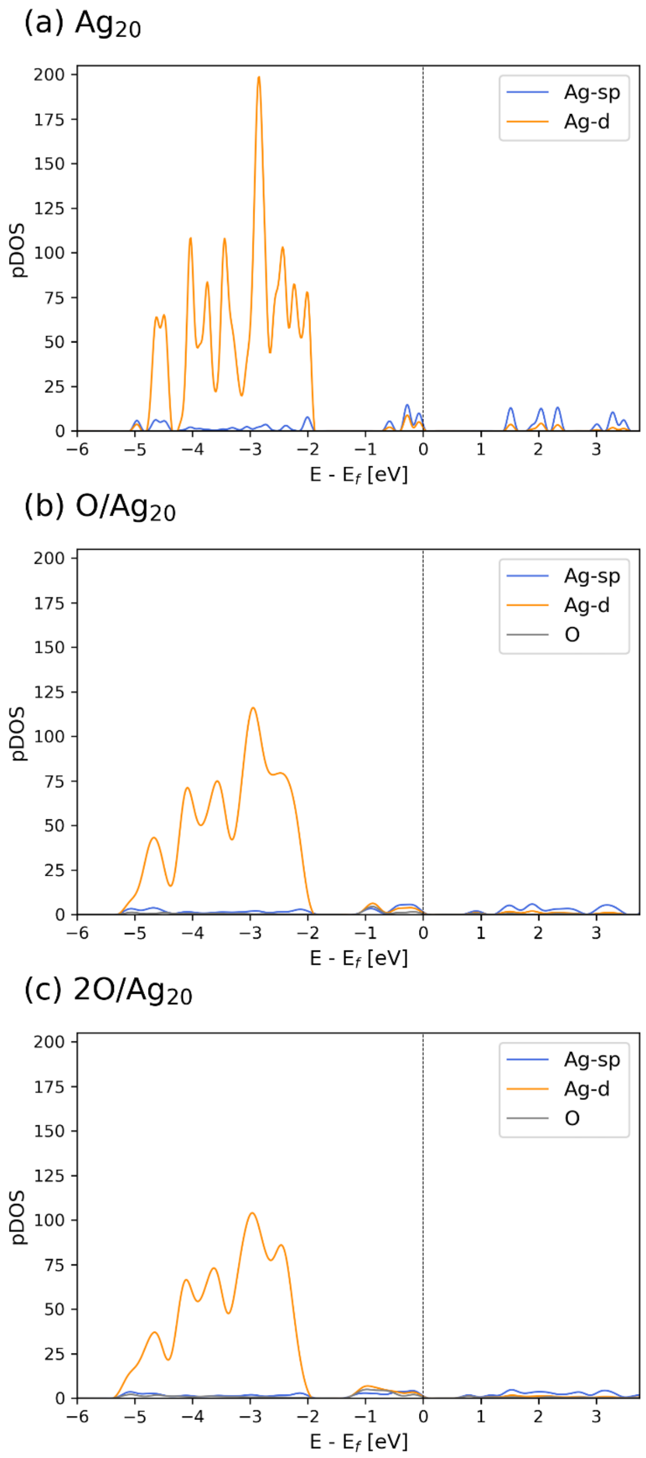

Figure S6: DOS projected on the *sp* or *d* states of the Ag_{20} (a), O/Ag_{20} (b), and $2O/Ag_{20}$ (c) systems illustrated in Figure 2a.

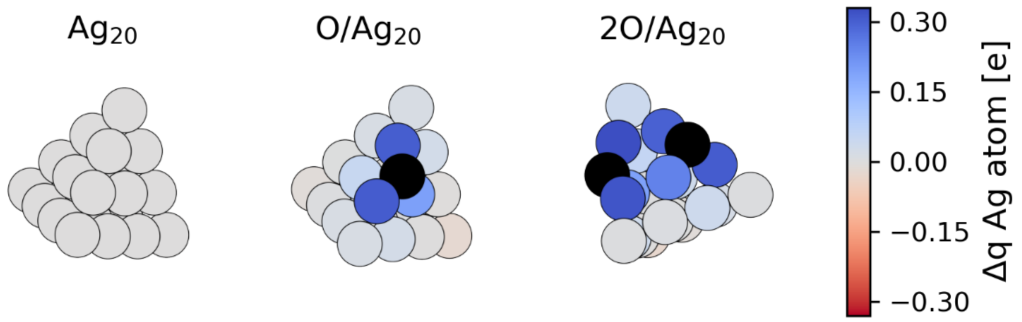

Figure S7: Bader charges of the Ag atoms in the Ag_{20}, O/Ag_{20} and $2O/Ag_{20}$ structures. The O atoms are coloured in black and have Bader charges of ~-0.9 |**e**|.

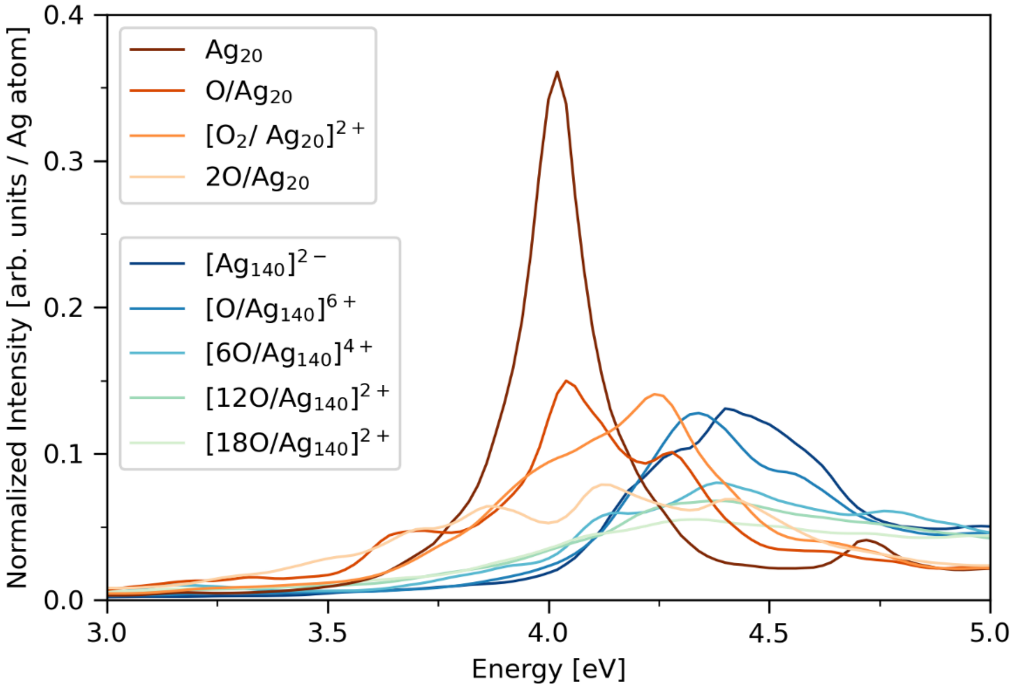

Figure S8: Photoabsorption spectra of Ag_{20}, O/Ag_{20}, $[O_2/Ag_{20}]^{2+}$, $2O/Ag_{20}$, $[Ag_{140}]^{2-}$, $[O/Ag_{140}]^{6+}$, $[6O/Ag_{140}]^{4+}$, $[12O/Ag_{140}]^{2+}$, and $[18O/Ag_{140}]^{2+}$ calculated with the polTDDFT method. Each spectrum has been normalized by the number of Ag atoms of the system.

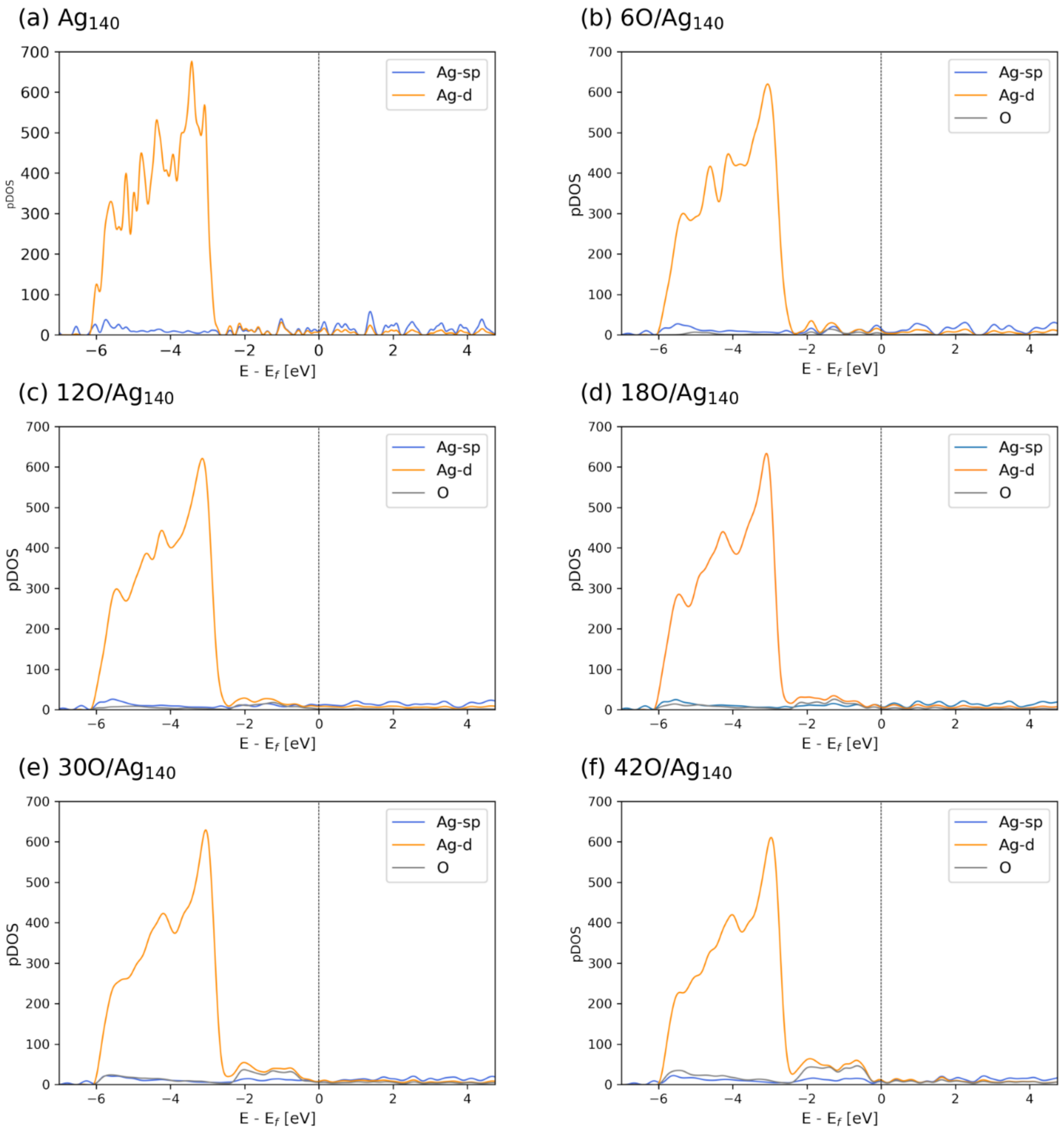

Figure S9: DOS projected on the *sp* or *d* states of Ag for the Ag_{140} (a), $6O/Ag_{140}$ (b), $12O/Ag_{140}$ (c), $18O/Ag_{140}$ (d), $30O/Ag_{140}$ (e), and $42O/Ag_{140}$ (f) systems illustrated in Figure 2b.

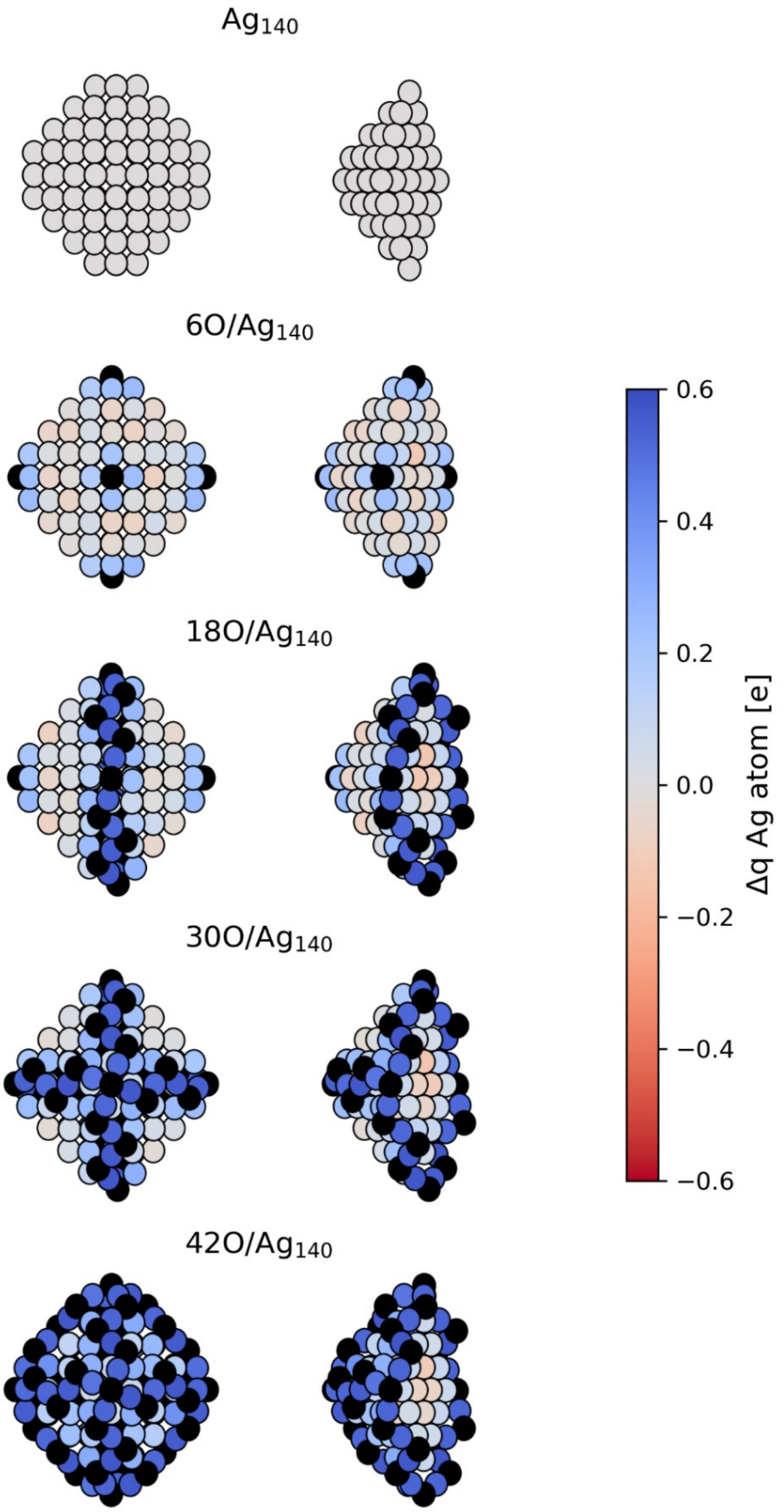

Figure S10: Bader charges of the Ag atoms in the Ag_{140}, $6O/Ag_{140}$, $18O/Ag_{140}$, $30O/Ag_{140}$, and $42O/Ag_{140}$ structures. The O atoms are coloured in black and have Bader charges of ~-0.9 |**e**|.
